# RKC-B1 Blocks Activation of NF-κB and NLRP3 Signaling Pathways to Suppress Neuroinflammation in LPS-Stimulated Mice

**DOI:** 10.3390/md19080429

**Published:** 2021-07-28

**Authors:** Man Liu, Ying-Lin Yang, Shan-Shan Zhang, Dong-Ni Liu, Lian-Hua Fang, Guan-Hua Du, Yue-Hua Wang

**Affiliations:** 1 State Key Laboratory of Bioactive Substance and Function of Natural Medicines, Institute of Materia Medica, Chinese Academy of Medical Sciences & Peking Union Medical College, Beijing 100050, China; liuman@imm.ac.cn (M.L.); yinglin@imm.ac.cn (Y.-L.Y.); zhangshanshan@imm.ac.cn (S.-S.Z.); liudongni@imm.ac.cn (D.-N.L.); dugh@imm.ac.cn (G.-H.D.); 2 Beijing Key Laboratory of Drug Target Identification and New Drug Screening, Institute of Materia Medica, Chinese Academy of Medical Sciences & Peking Union Medical College, Beijing 100050, China

**Keywords:** RKC-B1, neuroinflammation, lipopolysaccharide (LPS), NF-κB, NLRP3

## Abstract

RKC-B1 is a novel fermentation product obtained from the marine micromonospora FIM02-523A. Thus far, there have been few reports about the pharmacological activity of RKC-B1. In our present study, we investigated the anti-neuroinflammatory effects and the possible mechanism of RKC-B1 in LPS-stimulated mice. After treatment with RKC-B1, RNA-seq transcriptome of the cerebral cortex tissue was conducted to find the differentially expressed genes (DEGs). Inflammatory cytokines and proteins were evaluated by ELISA and WB. In RNA-seq analysis, there were 193 genes screened as core genes of RKC-B1 for treatment with neuroinflammation. The significant KEGG enrichment signaling pathways of these core genes were mainly included TNF signaling pathway, IL-17 signaling pathway, NOD-like receptor signaling pathway, NF-κB signaling pathway and others. The corresponding top five KEGG enrichment pathways of three main clusters in PPI network of core genes were closely related to human immune system and immune disease. The results showed that RKC-B1 reduced the levels of pro-inflammatory factors (IL-6, IL-1β, MCP-1, and ICAM-1) and the expression of COX2 in cerebral cortex tissue. Additionally, we found that the anti-neuroinflammation activity of RKC-B1 might be related to suppress activating of NF-κB and NLRP3/cleaved caspase-1 signaling pathways. The current findings suggested that RKC-B1 might be a promising anti-neuroinflammatory agent.

## 1. Introduction

Recently, neuroinflammation has been widely researched and seems to be a common feature to central nervous system (CNS) diseases, such as ischemic stroke (IS) [1], traumatic brain injury (TBI) [2], Parkinson’s disease (PD) [3], Alzheimer’s disease (AD) [4] and temporal lobe epilepsy (TLE) [5,6]. The activation of the neuroimmune cells, such as microglia and astrocytes, was one of the most characteristic events during the pathological process of neuroinflammation responses. Some studies have proved that neuroinflammation had both beneficial and detrimental consequences to the body. On the one hand, the inflammation in the CNS showed benefits on the proliferation and maturation of multiple neural precursor populations, regeneration of axon and the myelin reformation on denuded axons [7]. Besides, in a recent study, researchers have found that the increasing of several neurosteroids in activated microglia had protective effects for neuron [8]. On the other hand, aberrant inflammatory response was destructive to the health neurons in the brain through an increasing level of pro-inflammatory mediators, such as cytokines and ROS [9]. 

Microglia, as the first immunological barrier in the brain for defensing pathogens and environmental insults, play a crucial role in the central inflammatory response [10]. LPS, a common stimulus of inflammation, could trigger inflammatory responses in microglia via recognizing and binding its membrane receptors, Toll-like receptor (TLRs) [11]. Once the microglia were activated, various inflammatory mediators, such as interleukin-1 beta (IL-1β), interleukin-6 (IL-6) and tumor necrosis factor-alpha (TNF-α), would be released, which could further induce a wide scope of inflammatory responses [12]. After binding with LPS, TLR4 dimerizes following with MAPK signaling pathway and NF-κB signaling pathway initiated through interacting with downstream adaptor proteins, including MyD88-dependent and MyD88-independent pathways [13]. NF-κB activity was essential for immune responses and the activation of NF-κB signaling pathways was largely associated with inflammatory diseases [14,15]. Inflammasomes, a large intracellular multiprotein complex, were first described in detail in 2002. Among these inflammasomes discovered in recent years, NLRP3 inflammasome, which consists of three components, including NLRP3 scaffold, apoptosis-associated speck-like protein (ASC) and caspase-1 [16], was best characterized. Numerous reports have demonstrated that the dysregulation of NLRP3 inflammasome and the pathogenesis of several neuroinflammation diseases had strong relationship [17]. 

**Figure 1:**
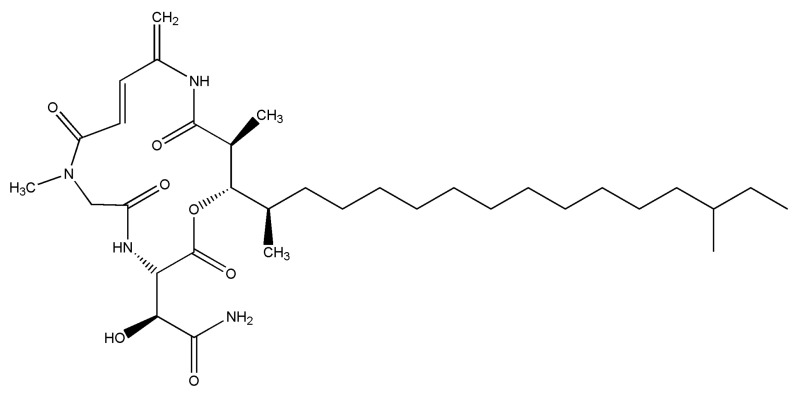
Structure information of RKC-B1 (C33H56N4O7, MW620.82).

RKC-B1 (Figure 1) was isolated from the marine micromonospora FIM02-523. Additionally, B1 single-component fermentation was obtained by high-yield strain breeding and optimization of fermenting. Nowadays, there were few reports on the pharmacological activity of RKC-B1 and the anti-neuroinflammation effects of RKC-B1 were unclear until now. Thus, in the present study, the anti-neuroinflammation effects and the possible underlying mechanisms of RKC-B1 in LPS-induced mice were investigated.

## 2. Results

### 2.1. Recognizing DEGs after RKC-B1 Treatment by RNA-Seq Transcriptome Analysis

**Figure 2:**
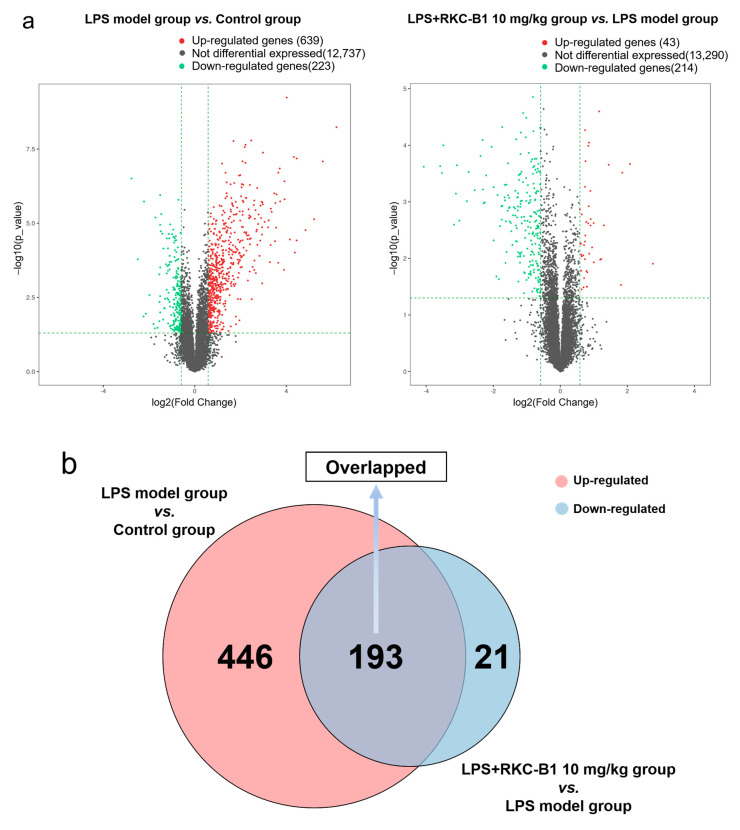

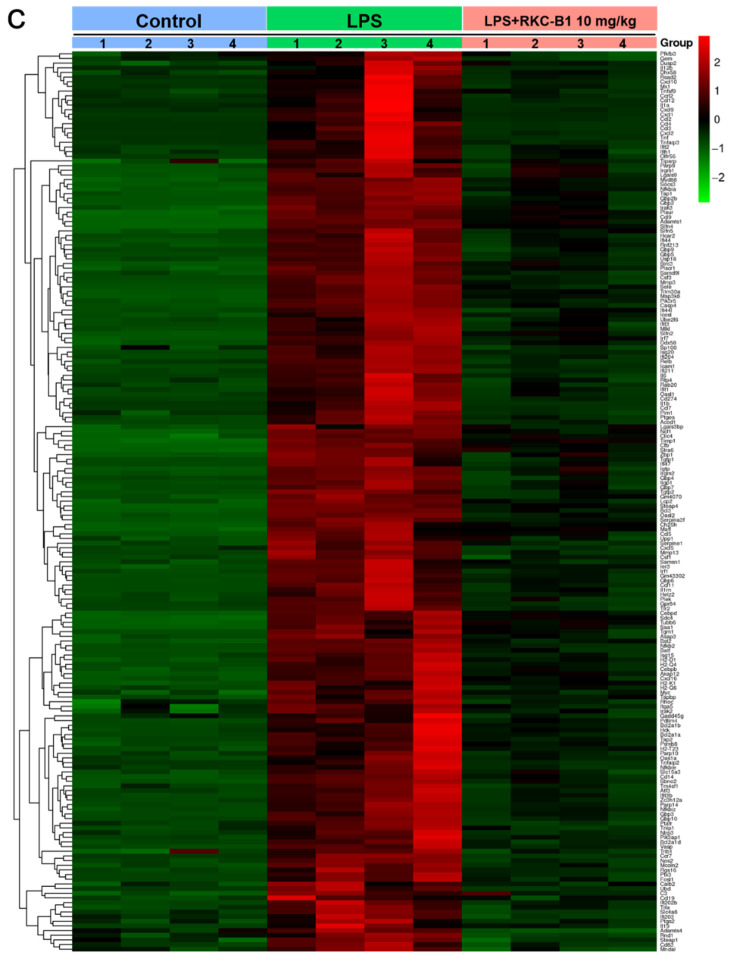
RNA sequencing analysis. (**a**) Volcano map analysis of DEGs in control group, LPS model group and LPS + RKC-B1 10 mg/kg group (*n* = 4); (**b**) Venn diagram of overlapped genes; (**c**) heat map of the overlapped genes. In this diagram, red means higher expression, and green means lower expression.

To explore the core targets for RKC-B1 against neuroinflammation induced by LPS, RNA sequencing analysis was carried out between LPS model group and control group and between LPS+RKC-B1 10 mg/kg group and LPS model group in this study. First, 639 differential up-regulated genes and 223 differential down-regulated genes were obtained between LPS model group and control group. Then, DEGs between RKC-B1 treated group and LPS model group were screened and 43 differential up-regulated genes and 214 differential down-regulated genes were found (Figure 2a). After drawing the Venn diagram, 193 genes were identified, which were up-regulated after LPS stimulating and down-regulated after RKC-B1 treatment (Figure 2b, Table S1). As shown in the heat map, the 193 overlapped genes were displayed by different colors among the control group, the LPS model group and the LPS + RKC-B1 10 mg/kg group (Figure 2c). In additional, these 193 overlapped genes were recognized as core genes for RKC-B1 against neuroinflammation, and then GO and KEGG pathway enrichment analysis were conducted.

### 2.2. GO and KEGG Enrichment Analysis of Core Genes for RKC-B1 against Neuroinflammation

**Figure 3:**
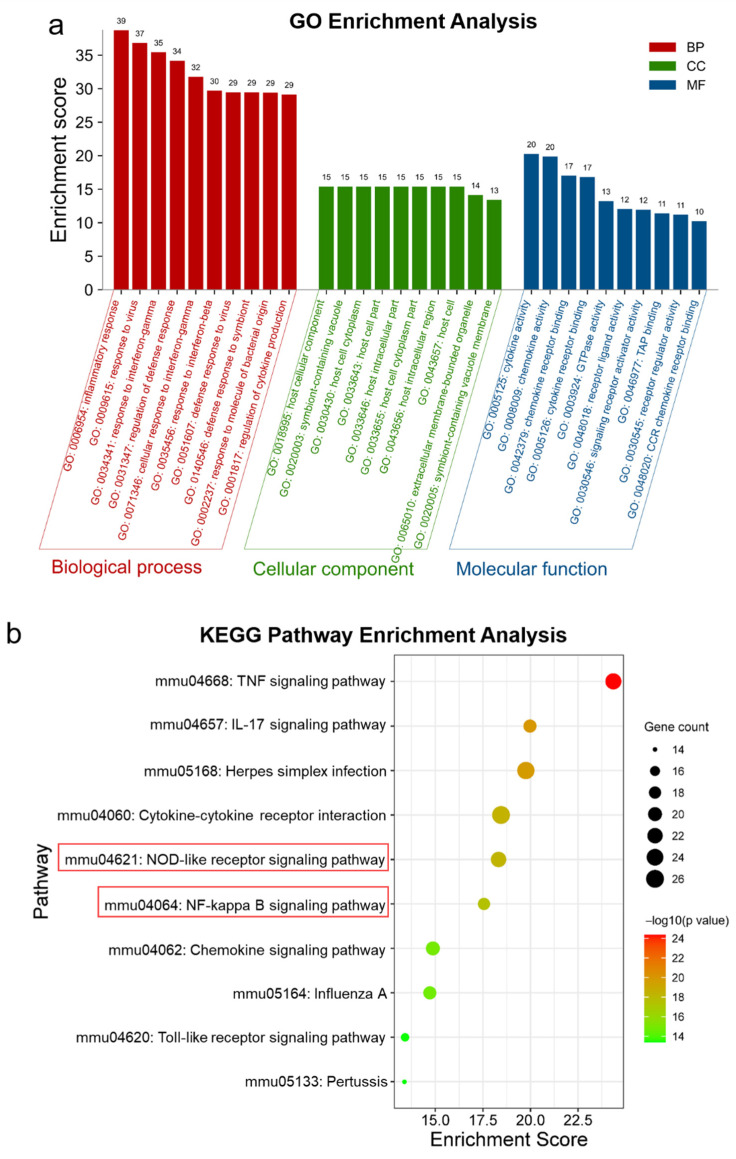

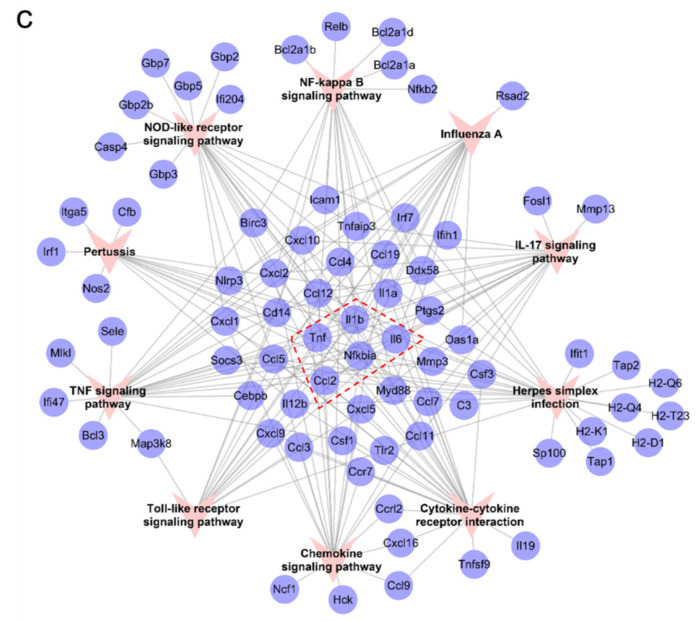
GO and KEGG enrichment analysis. (**a**) Top 10 GO terms, including BP, CC, and MF; (**b**) Top 10 KEGG pathways; (**c**) Targets–Pathway network of RKC-B1 against neuroinflammation.

To better understand the underlying anti-neuroinflammation mechanisms of RKC-B1, the mentioned 193 core genes were then processed with GO and KEGG enrichment analysis. Top ten significant GO terms and KEGG pathways were listed in Figure 3a,b. According to GO enrichment results, the biological process (BP) of anti-neuroinflammation effect for RKC-B1 were related to inflammatory response, response to virus, response to interferon-gamma, regulation of defense response and cellular response to interferon-gamma; the cellular component (CC) of anti-neuroinflammation effect for RKC-B1 were related to host cellular component, symbiont-containing vacuole, host cell cytoplasm, host cell part and host intracellular part; and the molecular function (MF) of anti-neuroinflammation effect for RKC-B1 included cytokine activity, chemokine activity, chemokine receptor binding, cytokine receptor binding and GTPase activity. Besides, the significant signaling pathways of KEGG enrichment analysis indicated that anti-neuroinflammation of RKC-B1 mainly included TNF signaling pathway, IL-17 signaling pathway, cytokine–cytokine receptor interaction, NF-κB signaling pathway, NOD-like receptor signaling pathway, chemokine signaling pathway and Toll-like receptor signaling pathway. Then, the targets-pathway network of RKC-B1 against neuroinflammation was constructed in Figure 3c. After analyzing the network, Tnf, Il1b, Nfkbia, Il6, Ccl2, Ccl12 and Ccl5 with higher degree value of 9, 9, 8, 8, 7, 7 and 7, respectively, among all the genes in the network might play a crucial role for RKC-B1 against neuroinflammation.

### 2.3. Identification of Main Clusters in PPI Network for RKC-B1 against Neuroinflammation

**Figure 4:**
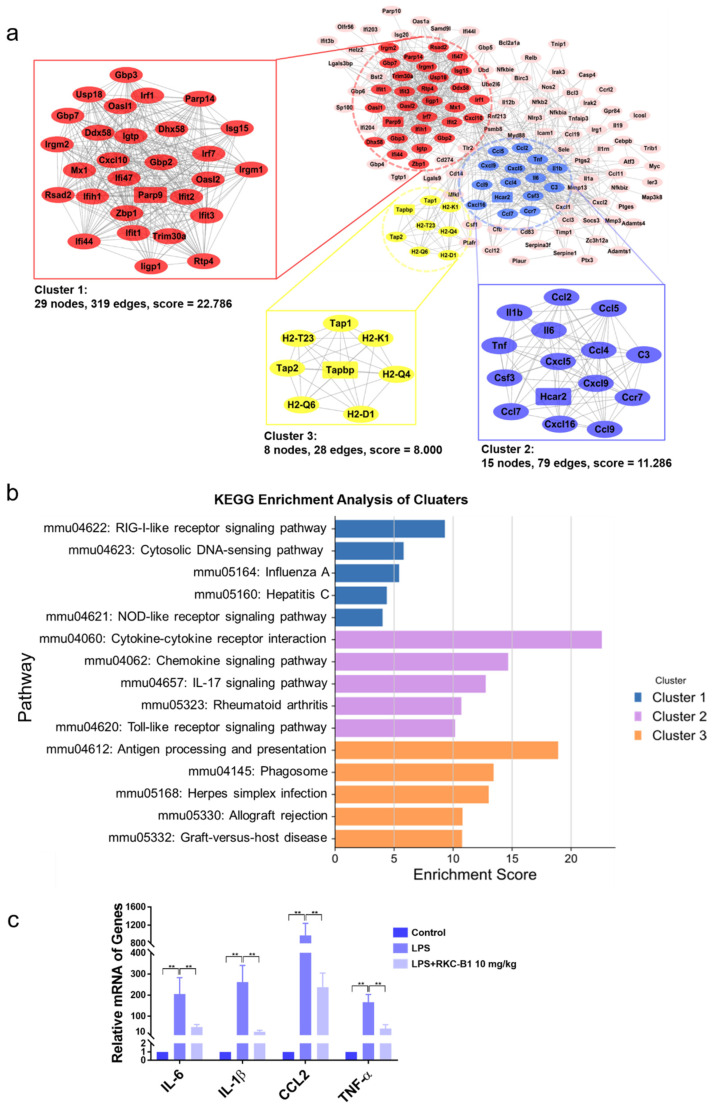
PPI network analysis of RKC-B1 against neuroinflammation. (**a**) PPI network and main clusters of RKC-B1 against neuroinflammation; (**b**) top five KEGG pathways of each cluster; (**c**) and relative mRNA level of IL-6, IL-1β, CCL2 and TNF-α (*n* = 5/group). Data are expressed as the mean ± SD. Statistical significance is assessed by one-way ANOVA analysis to compare the results between different groups ** *p* < 0.01 vs. LPS model group.

We further established the PPI network of the 193 core genes for RKC-B1 against neuroinflammation. After MCODE clustering analysis, the top 3 main PPI modules of PPI network were displayed as Figure 4a. Cluster 1 contained 29 nodes, 319 edges, a score of 22.786, and the seed gene (highest scoring node in the cluster) was Parp9; cluster 2 contained 15 nodes, 79 edges, a score of 11.286, and the seed gene was Hcar2; cluster 3 contained 8 nodes, 28 edges, a score of 8.000, and the seed gene was Tapbp. Then, we performed KEGG enrichment analysis to found the related pathways of these 3 clusters, respectively. Top five KEGG enrichment pathways of different clusters were listed as Figure 4b. In cluster 1, RIG-I-like receptor signaling pathway, cytosolic DNA-sensing pathway and NOD-like receptor signaling pathway belonged to “organismal systems, immune system” class, while influenza A and hepatitis C belonged to “human diseases, infectious disease: viral” class. In cluster 2, chemokine signaling pathway, IL-17 signaling pathway and Toll-like receptor signaling pathway belonged to “organismal systems, immune system” class, while rheumatoid arthritis belonged to “human diseases, immune disease” class. In cluster 3, antigen processing and presentation belonged to “organismal systems, immune system” class; herpes simplex infection belonged to “human diseases, infectious disease: viral” class; and allograft rejection and graft-versus-host disease belonged to “human diseases, immune disease” class. Therefore, the KEGG pathways of each cluster verified that the mechanisms for RKC-B1 against neuroinflammation were closely related to immune system and immune disease. Then, we conducted RT-PCR analysis of IL-6, IL-1β, CCL2 and TNF-α included in cluster 2 that were also ranked the top in the degree value in Figure 3c and associated with neuroinflammation as well-known pro-inflammatory factors. The RT-PCR results in Figure 4c were consistent with the RNA-Seq analysis.

### 2.4. RKC-B1 Decreased Pro-Inflammatory Factors and Mediators in Cortex of LPS-Stimulated Mice

**Figure 5:**
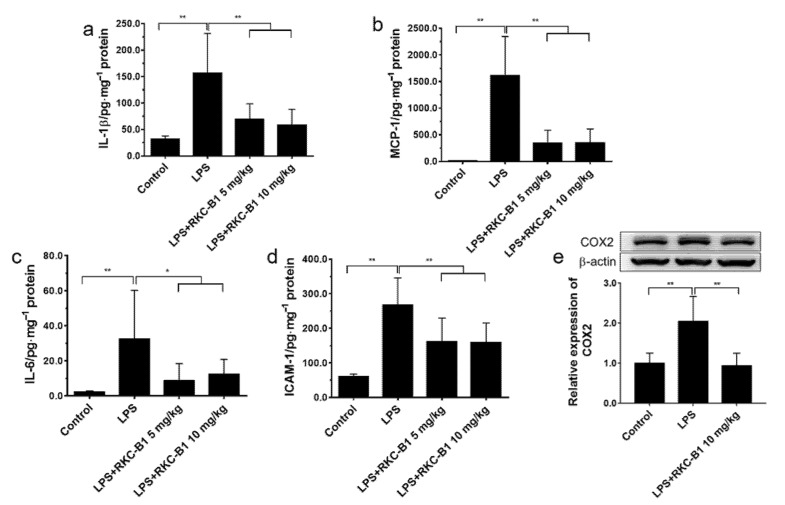
Effect of RKC-B1 on inflammatory factors in cortex tissue of LPS-stimulated mice. The levels of IL-1β (**a**), MCP-1 (**b**), IL-6 (**c**) and ICAM-1 (**d**) were assessed by ELISA (*n* = 7 or 8/group). The expression level of COX2 (**e**) were assessed by Western blot (*n* = 5/group). Data are expressed as the mean ± SD. Statistical significance is assessed by one-way ANOVA analysis to compare the results between different groups * *p* < 0.05, ** *p* < 0.01 vs. LPS model group.

To confirm the anti-inflammation effects of RKC-B1, the levels of pro-inflammatory factors, including IL-1β, IL-6, MCP-1 and ICAM-1 in cerebral cortex of mice were detected by ELISA (Figure 5a–d). In LPS model group, the levels of IL-1β, IL-6, MCP-1 and ICAM-1 were increased in the cerebral cortex. However, RKC-B1 at 5 and 10 mg/kg both significantly decreased the levels of IL-1β, IL-6, MCP-1 and ICAM-1 in cerebral cortex tissues. In addition, the level of COX2 was detected by WB (Figure 5e). The data indicated that the expression of COX2 was increased in the LPS model group, whereas RKC-B1 10 mg/kg significantly decreased the expressions of COX2.

### 2.5. RKC-B1 Inhibited the Phosphorylation of Major Proteins of NF-κB Signaling Pathway in Cortex of LPS-Stimulated Mice

**Figure 6:**
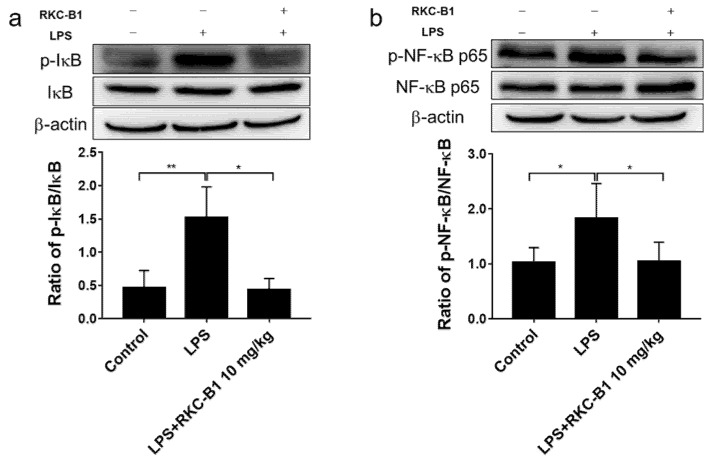
Effect of RKC-B1 on phosphorylation of IκB and NF-κB p65. (**a**) Ratio of p-IκB to IκB; (**b**) Ratio of p-NF-κB p65 to NF-κB p65; data are expressed as the mean ± SD (*n* = 5/group). Statistical significance is assessed by one-way ANOVA analysis to compare the results between different groups * *p* < 0.05, ** *p* < 0.01 vs. LPS model group.

According to the KEGG enrichment analysis of the core genes for RKC-B1 against neuroinflammation, we were strongly interested in the NF-κB signaling pathway. Besides, NF-κB signaling pathway was also a classical transduction pathway in the process of inflammation responses. Thus, we investigated the variation of protein expression for IκBα, phospho-IκBα, NF-κB p65 and phospho-NF-κB p65 by Western blot. Results showed that the ratios of p-IκBα/IκBα and p-NF-κB p65/NF-κB p65 were significantly increased after LPS stimulating. While RKC-B1 treatment inhibited the expression of phospho-NF-κB p65 and phospho-IκBα (Figure 6a,b).

### 2.6. RKC-B1 Inhibited the Translocation of NF-κB in Cortex of LPS-Stimulated Mice

**Figure 7:**
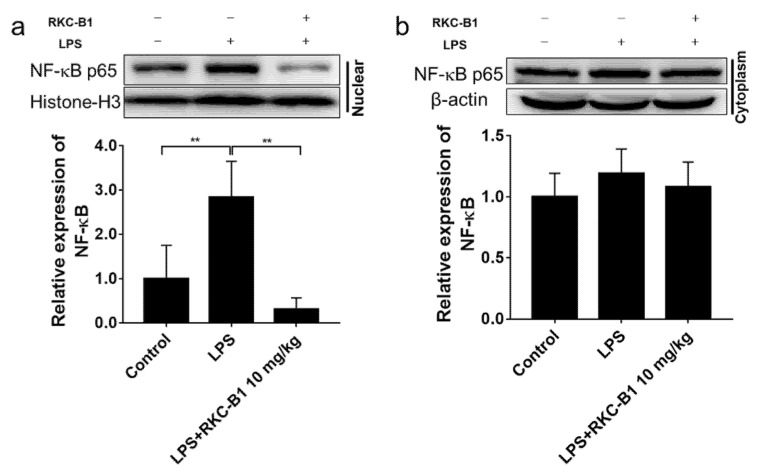
Effect of RKC-B1 on nuclear transposition of NF-κB p65. (**a**) The protein expression levels of NF-κB p65 in nuclear fraction; (**b**) the protein expression levels of NF-κB p65 in cytoplasmic fraction. Data are expressed as the mean ± SD (*n* = 5/group). Statistical significance is assessed by one-way ANOVA analysis to compare the results between different groups ** *p* < 0.01 vs. LPS model group.

Besides the phosphorylation of IκB and NF-κB p65 in NF-κB signaling pathways, we further investigated the NF-κB nuclear translocation in the present study. The results showed that the level of NF-κB p65 was significantly increased in the nucleus, but almost unchanged in cytoplasm after LPS injection in the cortex of mice. However, RKC-B1 treatment at 10 mg/kg notably suppressed NF-κB nuclear translocation (Figure 7a,b).

### 2.7. RKC-B1 Down-Regulated NLRP3/Cleaved Caspase-1 Signaling Pathway in LPS-Stimulated Mice

**Figure 8:**
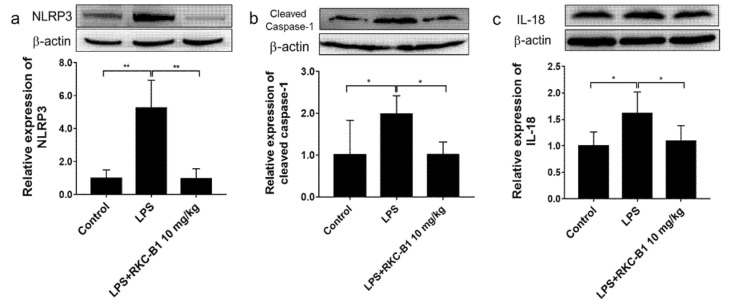
Effect of RKC-B1 on NLRP3/cleaved caspase-1 signaling pathway. (**a**) Protein expression levels of NLRP3; (**b**) protein expression levels of cleaved caspase-1; (**c**) protein expression levels of IL-18. Data are expressed as the mean ± SD (*n* = 5/group). Statistical significance is assessed by one-way ANOVA analysis to compare the results between different groups * *p* < 0.05, ** *p* < 0.01 vs. LPS model group.

NLRP3 was identified as core genes during RNA-seq analysis and NOD-like receptor signaling pathway emerged in KEGG enrichment analysis for RKC-B1 against neuroinflammation. Thus, we subsequently evaluated the major proteins expression levels of NLRP3/cleaved caspase-1 signaling pathway. We found that after LPS stimulating, the protein expression levels of NLRP3, cleaved caspase-1 and downstream IL-18 in the cortex of mice were significantly increased. However, 10 mg/kg RKC-B1 treatment inhibited the expression of NLRP3, cleaved caspase-1 and IL-18 (Figure 8a–c).

## 3. Discussion

RKC-B1, a kind of product of fermentation, was segregated from the marine micromonospora, FIM02-523. There was few information about the pharmacological activity of RKC-B1 due to its novelty. In our current study, we firstly proved that RKC-B1 has potential anti-neuroinflammation effect in the cerebral cortex tissue of LPS-induced mice. Besides, we found the protection of RKC-B1 against LPS-induced neuroinflammation might associate with blocking the activation of NF-κB and NLRP3 signaling pathways. 

Over the past 20 years, the development of RNA-seq has been flourishing and RNA-seq has become an omnipresent procedure in molecular biology [18]. By RNA-seq, differential gene expression (DGE) could be explored, which allows researchers to obtain the quantitative changes in expression levels of genes and (or) transcripts by comparing different experimental groups. In our present study, there were 639 up-regulated DGEs between the control group and the LPS model group and 214 down-regulated DGEs between the LPS model group and the RKC-B1 treatment group. Subsequently, we obtained 193 overlapped genes, which were regarded as the core genes for RKC-B1 against neuroinflammation. To further reveal the underlying association and meaning of these 193 core genes, GO and KEGG enrichment analysis and PPI analysis were carried out. The KEGG pathways for RKC-B1 against neuroinflammation included TNF signaling pathway, IL-17 signaling pathway, cytokine–cytokine receptor interaction, NF-κB signaling pathway, NOD-like receptor signaling pathway, chemokine signaling pathway and Toll-like receptor signaling pathway. TNF, as a critical cytokine involved in various diseases and is well known for its irreplaceable role in CSN signaling pathways [19]. TNF-α is usually associated with neurotoxicity. However, in some conditions it has been found to play a neuroprotective role [20]. The IL-17 family consisting of IL-17A-F, play an important role in both acute and chronic inflammation [21]. It has been reported that IL-17 signaling pathway could activate NF-κB and MAPK signaling pathways by binding their correspondent receptors that induced the expression of cytokines and chemokines [22]. Cytokines are produced by almost every cell to regulate immune response after insulting and the release of pro-inflammatory cytokines will lead to the activation of nearby immune cells and induce the production and release of further cytokines [23]. Chemokines are small secreted proteins that were known as chemotactic peptides to regulate the leukocytes trafficking. It is reported that chemokines and their receptors played an important role in the immune system [24]. LPS, as a kind of inflammation-inducing pathogen-associated molecular pattern (PAMP), could be recognized via pattern recognition receptors (PRRs), such as Toll-like receptors (TLR) and NOD-like receptors (NLR) [25]. Upon their activation, PRR transduces signals intracellularly via MAPK signaling pathways and NF-κB signaling pathways following with a cellular response [26]. 

Recently, the activating of microglia, the destruction of the blood–brain barrier, and the infiltration of peripheral immune cells in the brain have been demonstrated to be the characteristics of neuroinflammation [27]. Microglia activation would lead to product and release many pro-inflammatory factors, such as TNF-α, IL-1β, IL-6, etc. IL-1β and IL-6 are crucial pro-inflammatory cytokines that are produced by nearly every cell to regulate immune response and the activation of immune cells [23]. Monocyte chemoattractant protein (MCP-1/CCL2), one of the main chemokines, contributes to regulate the movement of microglial [28]. ICAM-1 is cell adhesion molecule and play an important role in the migration of leukocytes or monocytes to the infection site [29]. In the present study, the levels of IL-1β, IL-6, MCP-1 and ICAM-1 in cerebral cortex tissues were increased in LPS group compared with control group, which was consistent with the previous study [30]. However, RKC-B1 treatment decreased the levels of the inflammatory factors mentioned above in cerebral cortex tissue. COX2, as a crucial inflammatory-related enzyme, mediated the production of the pro-inflammatory factors, such as NO and PGE2, which aggravated the development of neuroinflammation [31]. Our results showed that LPS promoted the expression of COX2, while 10 mg/kg RKC-B1 reversed the increasing level of COX2 in the cerebral cortex tissue of LPS-induced mice.

TLR4 as the first-line host defense against invading pathogens on the surface of microglia leads to activation of multiple downstream signaling pathways such as NF-κB signaling pathways [32]. In normal circumstances, the NF-κB dimers p50 and p65 are inactive by binding with its inhibiting protein IκB in the cytoplasm. Upon activating, IκB is phosphorylated, ubiquitinated and finally degraded [33]. Subsequently, the activated NF-κB translocated into the nucleus where it bound to specific DNA binding sites. This led to transcriptional up-regulation of pro-inflammatory mediators [34]. It was reported that regulating the inflammatory environment benefited for improving the deficits and for inducing the endogenous ability of the brain to repair the damage [4]. In additional, numerous studies have found that blocking NF-κB signaling pathway could inhibit inflammatory responses [35,36]. In the present study, consistent with our previous findings, we observed that the expression of phosphorylated IκB and NF-κB were significantly increased in LPS group. While RKC-B1 treatment suppressed the phosphorylation of IκB and NF-κB. Moreover, we also found that RKC-B1 reversed the translocation of NF-κB induced by LPS. Taken together, the anti-neuroinflammation mechanism of RKC-B1 might be related to inhibit the activating of NF-κB signaling pathway.

IL-1β, functioning as a pro-inflammatory cytokine, plays crucial roles in inflammatory response. IL-18, as a co-factor in synergism with IL-12, exerts a potent pro-inflammatory effect via stimulating the production of gamma interferon (INF-γ) in Th1 cells [37]. Cleaved caspase-1, which could be activated by NLRP3 inflammasome, a large intracellular multiprotein complex containing caspase-1, ASC and NLRP3, is crucial for the transformation of IL-1β and IL-18 from precursor form to mature form, which could further trigger the downstream signaling pathways [38,39]. It has been reported that MCC950, a kind of the NLRP3 inflammasome inhibitor, could against neuroinflammation and had a neuroprotective effect which might be associated with reduced activation of microglial, recruitment of leukocyte, and production of pro-inflammatory cytokines [40,41]. Besides, researchers found that blocking NLRP3 function in vivo significantly delayed neuronal degeneration and ataxia onset in severe neurodegenerative diseases, such as cerebellar ataxias [42]. Thus, targeting NLRP3 signaling pathways for therapeutic strategy in human central nervous system pathologies has increasingly been a possibility of pharmacologically. In the present study, the levels of NLRP3, cleaved caspase-1, IL-1β and IL-18 were up-regulated after LPS stimulating. However, RKC-B1 treatment significantly reduced the levels of NLRP3, cleaved caspase-1, IL-1β and IL-18. Interestingly, NF-κB signaling pathway have been reported to participate in the priming step of NLRP3 inflammasome activating at the same time. The priming step initiated through PAMPs or DAMPs stimulating TLRs and subsequently resulted in the activation of NF-κB signaling pathway. Then, NF-κB induced transcription and the expression of NLRP3, pro-IL-1β and pro-IL-18 [17]. However, whether the activation of NLRP3 inflammasomes was inhibited via blocking the NF-κB-related priming step was still unknown in this study, which yet needed more in-depth studies in the future.

## 4. Materials and Methods

### 4.1. Reagents

RKC-B1 was kindly provided by Prof. Jian Zhou (Fujian Institute of Microbiology) and Prof. Hua-Wei Zhang (Zhejiang University of Technology). LPS (Escherichia coli 0127: B8) were purchased from Sigma Aldrich (St. Louis, MO, USA). ELISA kits IL-1β (EM001), IL-6 (EM004), MCP-1 (EM018) and ICAM-1 (EM013) were purchased from ExCell Biology (Shanghai, China). Antibodies for COX2 (ab15191) and IL-18 (ab207323) were purchased from Abcam (Cambridge, UK). Antibodies for β-actin (3700), NF-κB p65 (8242), phospho-NF-κB p65 (3033), IκBα (4814), phospho-IκBα (2859), NLRP3 (15101) and cleaved caspase-1 (89332) were purchased from Cell Signaling Technology (Beverley, CA, USA). 

### 4.2. Animals and Treatment

Adult male BALB/c mice with the weight of 28–22 g were purchased from SPF (Beijing, China) Biotechnology Co., Ltd. (Beijing, China; Animal certification number was SCXK (Jing) 2019–0010). All animals were kept in a pathogen-free condition with 12 h of light and dark per day at temperature of 23 ± 2 °C and humidity of 55 ± 5%. After acclimatizing for 3 days prior to experimentation, all animals were randomly divided into the following groups: control group (Control) (*n* = 12), which were pre-treated with an equal volume of vehicle by i.p. (normal saline with 0.1% Tween 80) for 7 days and normal saline by i.p. on the 7th day; LPS model group (LPS) (*n* = 12), which were pre-treated with an equal volume of vehicle by i.p. for 7 days and LPS (5 mg/kg) by i.p. on the 7th day; RKC-B1 treatment groups, which were pre-treated with RKC-B1 at doses of 5 mg/kg (LPS+ RKC-B1 5 mg/kg) (*n* = 8) and 10 mg/kg (LPS+ RKC-B1 10 mg/kg) (*n* = 12), respectively, by i.p. for 7 days and LPS (5 mg/kg) by i.p. on the 7th day [43]. After LPS injection for 6 h on the 7th day, the mice were sacrificed via decapitation and then the cortex tissues were removed and frozen at −80 °C for further research. Among the obtained cortex tissues of mice, 8 mice were used for ELISA, WB, and RT-PCR analysis, and 4 mice were used for RNA-Seq analysis. All animal care and experimental procedures regarding the animals were approved by the ethic committees of Institute of Materia Medica, Chinese Academy of Medical Sciences and Peking Union Medical College.

### 4.3. RNA-Seq Analysis

The cortex tissues of control group, LPS model group and LPS + RKC-B1 10 mg/kg group were processed for RNA-seq transcriptome analysis by using Hiseq4000 (Illumina, Hayward, CA, USA). The raw sequences were processed for quality control, trimming (Trimmomatic version 0.36) [44], and alignment (STAR/2.5.1b). Downstream analyses were performed by using R (version3.1.1, RStudio, Boston, MA, USA), with edgeR and limma package with voom method [45]. After the statistical analysis, p-values with the means of the two groups were calculated. Fold change of 1.5-fold (up or down) and p-value of 0.05 were used as a joint threshold to define biologically significant differences in gene expression. After obtaining the up-regulated DEGs (differential expression genes) through the comparison between LPS model group and control group and the down-regulated DEGs by the comparison between LPS+RKC-B1 10 mg/kg group and LPS model group. The overlapped up- and down-regulated DEGs were regarded as the core targets of RKC-B1 for treatment of neuroinflammation in this study. 

### 4.4. GO and KEGG Enrichment Analysis of the Core Targets for RKC-B1 against Neuroinflammation

Metascape (http://metascape.org, accessed on 19 May 2021) is a gene list analysis website which integrates more than forty bioinformatics knowledgebases and updates monthly [46]. It includes pathway enrichment analysis, protein interaction network structure analysis and rich gene annotation functions. In this study, the core targets for RKC-B1 against neuroinflammation were inputted into the Metascape database to conduct GO and KEGG enrichment analysis. *p* value ≤ 0.01 was considered significant. According to the p value, the top ten GO terms and top ten KEGG pathways were plotted in an online platform (http://www.bioinformatics.com.cn, accessed on 19 May 2021) for data visualization. Then, the target-pathway network of the core targets was diagramed by Cytoscape 3.8.0 (NIGMS, Bethesda, MD, USA).

### 4.5. Constructing PPI Network of the Core Targets for RKC-B1 against Neuroinflammation

Then, the core targets were introduced to an online platform String (https://string-db.org/, accessed on 20 May 2021) to obtain the interactions of these proteins [47]. As the following settings of the website, species chose “Mus Musculus”, minimum required interaction score chose “High confidence (0.700)”, and the disconnected nodes in the network chose “hided”. Then, the PPI network of the core targets was diagramed using Cytoscape 3.8.0 software.

### 4.6. Main Clusters of PPI Network Identification

MCODE, a cytoscape plugin, is often used to find clusters that are highly interconnected regions in a network [48]. Clusters mean different things in different types of networks. In a PPI network, clusters are often protein complexes and parts of pathways. In this study, clusters of PPI networks were obtained, and then the corresponding networks were analyzed by MCODE. Additionally, KEGG enrichment analysis for each of the top three clusters were carried out, respectively, to help us better understand the invisible meaning of each cluster in PPI network. 

### 4.7. ELISA Analysis

The supernatant samples of cortex tissues were prepared as described previously [43], and then the levels of IL-6, IL-1β, MCP-1, and ICAM-1 were detected by ELISA kits according to the introductions of the manufacturer.

### 4.8. Western Blot

After treatment with RKC-B1, the cortex tissues of mice were homogenized with cool RIPA (Radio Immunoprecipitation Assay) buffer with the cocktail protease inhibitor (Thermos, Waltham, MA, USA). The homogenization was kept on the ice for 30 min and then centrifuged at 12,000× *g* at 4 °C for 15 min. The supernatants were then taken apart and the protein concentration was determined by BCA assay kit (Thermos, Waltham, MA, USA). Then, 5× loading buffer was added into samples and then boiled the samples for 10 min at 100 °C. Total protein was separated by 10~15% SDS-PAGE, and the proteins were transferred to polyvinylidene fluoride (PVDF) membrane. The membranes were incubated with 5% bovine serum albumin (BSA) for 2 h at the temperature of 25 °C and then incubated with the following different primary antibodies: anti-COX2 antibody (Rt, 1:1000), anti-NLRP3 antibody (Rt, 1:1000), anti-cleaved caspase-1 antibody (Rt, 1:1000), anti-IL-18 antibody (Rb, 1:1000), anti-NF-κB p65 antibody (Rb, 1:1000), anti-phospho-NF-κB p65 antibody (Rb, 1:1000), anti-IκBα antibody (Ms, 1:1000), anti-phospho-IκBα antibody (Rb, 1:1000) and anti-β-actin antibody (Ms, 1:3000) at 4 °C overnight. The primary antibodies were washed out using 1×TBST and then the membranes were incubated with HRP-conjugated secondary antibody for 2 h at room temperature. After washing by 1×TBST the membranes were finally detected by enhanced ECL system. The absolute integrated OD of each band were measured by Gel-pro software (Molecular Imager ChemiDoc XRS + System, Bio-Rad, CA, USA).

### 4.9. Real-Time PCR Analysis

Total RNA was extracted from cortex tissues of BALB/c mice using Trizol reagent (Ambion, Carlsbad, CA, USA) according to the manufacturer’s instructions. The RNA samples (1000 ng/μL) were used for reverse transcription with MonScript^TM^RTⅢ All-in-One Mix (Monad Biotech Co., Ltd., Wuhan, China). The primers included β-actin (forward, AGGCCAACCGTGAAAAGATG; reverse, TGGCGTGAGGGAGAGCATAG), IL-6 (forward, CCACTTCACAAGTCGGAGGCTTA; reverse, GCAAGTGCATCATCGTTGTTCATAC), IL-1β (forward, CCAGGATGAGGACATGAGCA; reverse, CGGAGCCTGTAGTGCAGTTG) and TNF-α (forward, CCACGCTCTTCTGTCTACTG; reverse, ACTTGGTGGTTTGCTACGAC) and CCL2 (forward, AGCACCAGCACCAGCCAACT; reverse, CAGGTGACTGGGGCATTGAT). Then, according to the primers, 2 μL cDNA templates were subjected to real-time PCR using SYBR^®^ qPCR Master Mix (Vazyme Biotech Co., Ltd., Nanjing, China). The protocol of RT-PCR and calculation method were the same as previous described [49].

### 4.10. Statistical Analysis

Statistical analysis was performed using GraphPad Prism7 software (GraphPad Software, San Diego, CA, USA). Data were presented as mean ± SD in this study. The data were analyzed by one-way analysis of variance (ANOVA) followed by Dunnett’s multiple comparisons test. *p* < 0.05 was considered to be statistically significant.

## 5. Conclusions

In summary, in the present study, we found RKC-B1 markedly suppressed the levels of inflammatory factors and inflammatory proteins of LPS-induced neuroinflammation injury in mice. Besides, the mechanism of anti-neuroinflammation might associate with blocking NF-κB and NLRP3/cleaved caspase-1 signaling pathways. However, more studies need to be carried out, and different specific inhibitors would be used to explore the in-depth mechanism in the future study. Overall, the results in the study showed that RKC-B1 might be a potential anti-neuroinflammatory agent.

## Supplementary Materials

The following are available online at https://www.mdpi.com/article/10.3390/md19080429/s1, Table S1 A list of 193 core genes regulated by RKC B1 in LPS stimulated mice.
