# Supplementary material for: RKC-B1 Blocks Activation of NF-κB and NLRP3 Signaling Pathways to Suppress Neuroinflammation in LPS-Stimulated Mice
Source: Mar Drugs. 2021 Jul 28;19(8):429. doi: 10.3390/md19080429 (PMC8398414; doi:10.3390/md19080429)
Supplement: Supplementary file 1 [file marinedrugs-19-00429-s001.zip › marinedrugs-1319041-SI.pdf]

# Supplementary information

## RKC-B1 blocks activation of NF-κB and NLRP3 signaling pathways to suppress neuroinflammation in LPS-stimulated mice

**Table S1 A list of 193 core genes regulated by RKC-B1 in LPS-stimulated mice.**

| | Gene Name | LPS vs Control | | | LPS+RKC-B1 vs LPS | | |
|---|---|---|---|---|---|---|---|
| | | Fold Change | p value | q value | Fold Change | p value | q value |
| **1** | Cxcl1 | 65.10088711 | 4.26E-06 | 0.00257843 | 0.09892440 | 2.00E-04 | 0.15323624 |
| **2** | Ccl2 | 36.07034489 | 9.61E-06 | 0.00405492 | 0.07387910 | 3.13E-04 | 0.17615244 |
| **3** | Csf3 | 21.92767239 | 6.56E-08 | 0.00026853 | 0.19249480 | 1.55E-04 | 0.15281927 |
| **4** | Cxcl10 | 43.97913935 | 9.25E-06 | 0.00395844 | 0.07993435 | 4.96E-03 | 0.55866166 |
| **5** | Ccl3 | 20.77689607 | 9.76E-05 | 0.01733359 | 0.05945455 | 2.40E-04 | 0.16572972 |
| **6** | Gbp2b | 20.10588909 | 5.87E-08 | 0.00026853 | 0.30241717 | 1.18E-03 | 0.31233558 |
| **7** | Il1b | 23.53075864 | 2.34E-05 | 0.00692465 | 0.07644787 | 7.96E-05 | 0.14107387 |
| **8** | Steap4 | 16.21418317 | 5.83E-10 | 0.00001311 | 0.43486529 | 3.23E-04 | 0.17615244 |
| **9** | Timp1 | 13.72121788 | 3.59E-06 | 0.00233615 | 0.44582941 | 1.71E-02 | 0.81512380 |
| **10** | Ccl12 | 14.55324950 | 3.20E-04 | 0.03776093 | 0.12764284 | 1.96E-03 | 0.39419250 |
| **11** | Gbp2 | 13.18838336 | 1.42E-07 | 0.00042659 | 0.35084737 | 1.20E-03 | 0.31233558 |
| **12** | Iigp1 | 11.32445709 | 2.27E-06 | 0.00184033 | 0.20359328 | 8.68E-03 | 0.67005142 |
| **13** | Ccl4 | 12.54038353 | 2.15E-04 | 0.02989038 | 0.08667578 | 3.10E-04 | 0.17615244 |
| **14** | Socs3 | 12.68089101 | 1.02E-06 | 0.00135136 | 0.39122090 | 6.52E-04 | 0.24706111 |
| **15** | Ccl5 | 18.43246597 | 8.71E-06 | 0.00389854 | 0.40116579 | 2.70E-02 | 0.85946297 |
| **16** | Cd14 | 11.50002386 | 2.09E-06 | 0.00184033 | 0.34704313 | 8.52E-04 | 0.27125609 |
| **17** | Ccl7 | 11.36488072 | 4.03E-05 | 0.01005075 | 0.15049389 | 2.97E-04 | 0.17615244 |
| **18** | Gbp5 | 13.40717907 | 2.47E-05 | 0.00711916 | 0.27925266 | 1.52E-03 | 0.35884891 |
| **19** | Icam1 | 14.03481743 | 2.25E-06 | 0.00184033 | 0.21992088 | 8.88E-05 | 0.14107387 |
| **20** | Isg15 | 9.98986670 | 5.44E-05 | 0.01182828 | 0.27317044 | 7.59E-04 | 0.25970400 |
| **21** | Cxcl2 | 13.7594041 | 1.91E-04 | 0.02776600 | 0.10404587 | 1.53E-03 | 0.35884891 |
| **22** | Ptx3 | 12.3252538 | 5.34E-06 | 0.00288902 | 0.17852838 | 1.39E-03 | 0.34008544 |
| **23** | Gbp3 | 13.0800332 | 4.33E-05 | 0.01039623 | 0.09381898 | 8.20E-05 | 0.14107387 |
| **24** | Gpr84 | 8.31931429 | 9.05E-06 | 0.00394549 | 0.20087584 | 8.07E-05 | 0.14107387 |
| **25** | Gbp4 | 8.93572584 | 2.36E-03 | 0.12517640 | 0.09540324 | 1.14E-03 | 0.30775113 |
| **26** | Cebpd | 7.83648667 | 2.38E-06 | 0.00187519 | 0.50613002 | 2.20E-03 | 0.42179550 |
| **27** | Tlr2 | 7.78292299 | 1.99E-06 | 0.00184033 | 0.21598554 | 3.41E-04 | 0.18210769 |
| **28** | Il1a | 7.71473463 | 1.06E-03 | 0.07803015 | 0.11566249 | 7.15E-04 | 0.25445205 |
| **29** | Tgtp1 | 8.31948885 | 1.13E-05 | 0.00431908 | 0.27909433 | 2.79E-03 | 0.45356546 |
| **30** | H2-Q4 | 9.82792199 | 3.58E-05 | 0.00934001 | 0.33683376 | 1.79E-03 | 0.38267144 |
| **31** | Bst2 | 7.86512774 | 2.14E-06 | 0.00184033 | 0.41799284 | 3.19E-04 | 0.17615244 |
| **32** | Akap12 | 7.49198750 | 6.64E-06 | 0.00328040 | 0.48610759 | 3.21E-03 | 0.47187986 |

| | | | | | | | |
|---|---|---|---|---|---|---|---|
| **33** | Gm43302 | 7.31334956 | 2.55E-06 | 0.00194333 | 0.32860608 | 4.97E-04 | 0.22067291 |
| **34** | Ccl11 | 6.90826646 | 5.09E-06 | 0.00287188 | 0.31851174 | 9.43E-04 | 0.28426970 |
| **35** | Ifit3 | 6.66148571 | 5.34E-05 | 0.01169495 | 0.32205890 | 1.20E-02 | 0.73162649 |
| **36** | Tgtp2 | 6.79510967 | 6.27E-07 | 0.00100798 | 0.31526440 | 5.08E-04 | 0.22139172 |
| **37** | Ifi202b | 6.56649646 | 1.64E-05 | 0.00553130 | 0.29551771 | 1.20E-03 | 0.31233558 |
| **38** | Ch25h | 6.48160714 | 1.90E-05 | 0.00613844 | 0.56923262 | 1.32E-02 | 0.74846184 |
| **39** | Tnf | 6.36161560 | 4.79E-04 | 0.04949381 | 0.17057795 | 6.22E-04 | 0.24487852 |
| **40** | Ifit1 | 6.10879809 | 1.80E-04 | 0.02654182 | 0.32904632 | 7.68E-03 | 0.64959760 |
| **41** | Mmp3 | 6.07849837 | 1.72E-06 | 0.00181909 | 0.34001259 | 7.64E-04 | 0.25970400 |
| **42** | Tnfaip2 | 5.53540847 | 1.94E-04 | 0.02798755 | 0.20869029 | 4.75E-04 | 0.21980543 |
| **43** | Bcl2a1b | 5.79363935 | 2.95E-04 | 0.03665867 | 0.41657775 | 9.26E-03 | 0.67398655 |
| **44** | Rsad2 | 6.47049909 | 1.64E-03 | 0.10124290 | 0.31363450 | 2.78E-02 | 0.86073554 |
| **45** | Igtp | 4.72976861 | 4.71E-07 | 0.00091939 | 0.43132019 | 1.69E-02 | 0.81512380 |
| **46** | Hcar2 | 5.42895462 | 3.23E-05 | 0.00872614 | 0.35186341 | 2.21E-03 | 0.42179550 |
| **47** | Slfn2 | 5.29151478 | 3.41E-05 | 0.00910964 | 0.52459165 | 1.40E-02 | 0.76617254 |
| **48** | Irgm1 | 8.89078703 | 3.02E-04 | 0.03709785 | 0.46707307 | 8.72E-03 | 0.67005142 |
| **49** | Nfkbia | 5.28491057 | 6.72E-06 | 0.00328040 | 0.50980116 | 5.55E-04 | 0.23067121 |
| **50** | Il1rn | 9.90761778 | 1.43E-04 | 0.02306416 | 0.30982611 | 2.77E-02 | 0.86073554 |
| **51** | Gbp6 | 4.95980316 | 3.44E-05 | 0.00316343 | 0.40082840 | 2.62E-03 | 0.18707651 |
| **52** | Maff | 5.15505586 | 8.67E-05 | 0.01583502 | 0.52770584 | 1.07E-02 | 0.71840014 |
| **53** | Plaur | 5.99847762 | 7.08E-05 | 0.01419552 | 0.46917299 | 2.22E-02 | 0.84534535 |
| **54** | Tubb6 | 4.72713498 | 2.23E-05 | 0.00677355 | 0.62979200 | 1.61E-02 | 0.81510096 |
| **55** | Adamts1 | 4.81660198 | 7.17E-08 | 0.00026853 | 0.57777295 | 1.62E-03 | 0.36300658 |
| **56** | Cfb | 3.59496597 | 6.44E-04 | 0.05807775 | 0.57582370 | 4.77E-04 | 0.10849138 |
| **57** | Bcl3 | 4.81677618 | 6.25E-07 | 0.00100798 | 0.44954624 | 9.75E-05 | 0.14107387 |
| **58** | Cd274 | 4.41255286 | 1.18E-04 | 0.02003477 | 0.31614637 | 8.33E-04 | 0.27125609 |
| **59** | Ifi47 | 4.59699629 | 1.49E-03 | 0.09562894 | 0.20816392 | 2.31E-03 | 0.42461409 |
| **60** | Sdc4 | 4.37806811 | 1.92E-06 | 0.00184033 | 0.62529919 | 1.45E-03 | 0.35002109 |
| **61** | Gbp10 | 4.42076404 | 6.51E-06 | 0.00325125 | 0.33065353 | 2.34E-04 | 0.16556984 |
| **62** | Saa1 | 11.4704569 | 3.39E-06 | 0.00229302 | 0.32369748 | 2.93E-03 | 0.45908435 |
| **63** | Ifit3b | 4.24914411 | 3.77E-05 | 0.00963045 | 0.37631837 | 1.73E-03 | 0.37818860 |
| **64** | Ccl9 | 4.26565053 | 1.02E-07 | 0.00034611 | 0.59836897 | 3.10E-04 | 0.17615244 |
| **65** | Ier3 | 4.20437376 | 1.29E-04 | 0.02138703 | 0.51422708 | 2.75E-03 | 0.45247395 |
| **66** | Irf7 | 3.46992210 | 5.01E-04 | 0.05012260 | 0.43545007 | 1.92E-03 | 0.39419250 |
| **67** | Bcl2a1a | 4.17215427 | 2.97E-04 | 0.03673555 | 0.40759429 | 5.01E-03 | 0.55866166 |
| **68** | Stra6 | 4.15964635 | 4.79E-07 | 0.00027906 | 0.60547826 | 3.72E-03 | 0.22611303 |
| **69** | Oasl2 | 5.26312875 | 6.06E-06 | 0.00316439 | 0.37566464 | 7.29E-05 | 0.14107387 |
| **70** | Gbp9 | 4.00241803 | 3.95E-05 | 0.00336796 | 0.36351594 | 3.30E-03 | 0.21915264 |
| **71** | Cebpb | 3.98312654 | 1.24E-04 | 0.02078305 | 0.50562021 | 6.71E-03 | 0.61316241 |
| **72** | Slfn4 | 3.85467747 | 6.51E-07 | 0.00100798 | 0.44460372 | 3.18E-03 | 0.47187986 |
| **73** | Gbp7 | 3.26367856 | 3.30E-03 | 0.15229110 | 0.44977299 | 4.00E-02 | 0.90083858 |
| **74** | Nfkb2 | 4.58342779 | 2.92E-04 | 0.03650130 | 0.38781780 | 6.97E-05 | 0.14107387 |

| | | | | | | | |
|---|---|---|---|---|---|---|---|
| **75** | Atf3 | 4.07663701 | 1.50E-04 | 0.02404740 | 0.32867098 | 1.36E-03 | 0.33732611 |
| **76** | Bcl2a1d | 3.90237422 | 1.01E-04 | 0.01785452 | 0.38225536 | 1.24E-03 | 0.31688803 |
| **77** | Il12b | 3.94196204 | 2.13E-02 | 0.39389411 | 0.25368078 | 2.13E-02 | 0.84390998 |
| **78** | Parp14 | 4.39147462 | 5.87E-05 | 0.01244262 | 0.37216639 | 9.76E-04 | 0.28426970 |
| **79** | Nfkbiz | 3.83825953 | 1.04E-04 | 0.00635180 | 0.44020464 | 1.09E-03 | 0.13780588 |
| **80** | Irgm2 | 3.04841227 | 2.18E-06 | 0.00184033 | 0.52726994 | 2.72E-02 | 0.85946297 |
| **81** | Irf1 | 3.13707838 | 2.03E-02 | 0.38971307 | 0.35542414 | 1.44E-02 | 0.77111897 |
| **82** | Trim30a | 3.68998818 | 4.68E-06 | 0.00273124 | 0.53278649 | 8.92E-04 | 0.27429079 |
| **83** | Isg20 | 3.05368838 | 1.26E-05 | 0.00462809 | 0.48732751 | 9.01E-05 | 0.14107387 |
| **84** | Tnfaip3 | 4.46648211 | 2.02E-03 | 0.11477613 | 0.25466278 | 4.16E-03 | 0.51231447 |
| **85** | Ptges | 3.44657866 | 1.08E-04 | 0.01873055 | 0.41033202 | 9.61E-04 | 0.28426970 |
| **86** | Ifi44 | 5.39591097 | 7.56E-06 | 0.00353620 | 0.39284430 | 2.45E-03 | 0.43704726 |
| **87** | Oas1a | 3.32962654 | 1.31E-03 | 0.08939661 | 0.44797941 | 7.80E-03 | 0.64959760 |
| **88** | Usp18 | 4.32880016 | 7.02E-05 | 0.01419552 | 0.38031788 | 1.19E-03 | 0.31233558 |
| **89** | Birc3 | 4.20003971 | 4.82E-06 | 0.00277471 | 0.52752270 | 4.29E-03 | 0.51883193 |
| **90** | Hck | 2.39965044 | 2.12E-03 | 0.11775856 | 0.41512996 | 1.05E-02 | 0.71492553 |
| **91** | Rgs16 | 3.22668854 | 2.15E-04 | 0.02989038 | 0.41428675 | 8.24E-04 | 0.27125609 |
| **92** | H2-D1 | 3.23396014 | 9.10E-05 | 0.01647956 | 0.50377041 | 1.85E-02 | 0.81944133 |
| **93** | Cxcl16 | 3.09109168 | 2.75E-04 | 0.03485603 | 0.56405344 | 1.35E-02 | 0.76078256 |
| **94** | Pik3r5 | 3.74453775 | 1.27E-04 | 0.02103875 | 0.46617270 | 5.65E-05 | 0.12678100 |
| **95** | Rab20 | 3.11072761 | 2.37E-04 | 0.03155199 | 0.40943568 | 1.88E-03 | 0.39419250 |
| **96** | Casp4 | 4.26130153 | 2.07E-05 | 0.00652485 | 0.45538012 | 1.70E-03 | 0.37498510 |
| **97** | Plscr1 | 3.18293107 | 3.77E-05 | 0.00963045 | 0.62481451 | 5.69E-03 | 0.58439418 |
| **98** | Slc4a8 | 3.12247536 | 1.64E-04 | 0.02520593 | 0.29275952 | 5.48E-05 | 0.12678100 |
| **99** | H2-K1 | 3.12990095 | 3.40E-04 | 0.03931408 | 0.63462584 | 1.65E-02 | 0.81512380 |
| **100** | Sbno2 | 3.20783816 | 5.05E-03 | 0.19519900 | 0.32211449 | 4.86E-03 | 0.55130179 |
| **101** | Serpine1 | 3.20244686 | 3.69E-05 | 0.00951981 | 0.43024199 | 4.34E-04 | 0.20484491 |
| **102** | Zc3h12a | 3.09005963 | 1.56E-04 | 0.02467952 | 0.43510604 | 1.48E-03 | 0.35274553 |
| **103** | Ifit2 | 2.98072997 | 1.14E-03 | 0.08203928 | 0.43522106 | 8.72E-03 | 0.67005142 |
| **104** | Ncf1 | 2.07112041 | 1.09E-02 | 0.29610407 | 0.46886151 | 3.01E-03 | 0.46350090 |
| **105** | Sele | 2.95338707 | 1.45E-05 | 0.00518752 | 0.53325947 | 1.02E-03 | 0.29191758 |
| **106** | Tgm1 | 2.56995608 | 1.29E-03 | 0.08858205 | 0.54012280 | 1.70E-02 | 0.46700372 |
| **107** | Ifi204 | 3.86835701 | 4.82E-05 | 0.01111588 | 0.38512947 | 8.93E-04 | 0.27429079 |
| **108** | Ifi44l | 2.85401390 | 5.11E-05 | 0.01142079 | 0.48385645 | 4.34E-03 | 0.51883193 |
| **109** | Lgals9 | 2.33644393 | 1.79E-04 | 0.02638974 | 0.61700223 | 3.74E-02 | 0.61935777 |
| **110** | Oasl1 | 2.80456202 | 1.63E-04 | 0.02520593 | 0.47244096 | 9.05E-03 | 0.34450692 |
| **111** | Myd88 | 2.54921706 | 3.08E-05 | 0.00847912 | 0.58461497 | 2.31E-04 | 0.08859492 |
| **112** | Psmb8 | 3.11461269 | 1.09E-03 | 0.07984665 | 0.57990474 | 2.64E-02 | 0.85831106 |
| **113** | Irak3 | 2.75054117 | 1.41E-05 | 0.00181443 | 0.59636038 | 5.51E-03 | 0.27900476 |
| **114** | Nfkbie | 2.74885662 | 1.66E-03 | 0.10209931 | 0.40732690 | 2.94E-03 | 0.45908435 |
| **115** | Lgals3bp | 3.03818042 | 1.32E-02 | 0.32410880 | 0.62643182 | 3.97E-02 | 0.63487012 |
| **116** | Tap2 | 2.71821288 | 4.95E-04 | 0.01814413 | 0.52490773 | 3.65E-03 | 0.22611303 |

| | | | | | | | |
|---|---|---|---|---|---|---|---|
| **117** | Serpina3f | 2.85090142 | 2.97E-07 | 0.00066762 | 0.39693678 | 4.03E-04 | 0.20069936 |
| **118** | Clic4 | 2.66928964 | 2.17E-05 | 0.00666797 | 0.65792288 | 3.16E-04 | 0.17615244 |
| **119** | Map3k8 | 2.64109995 | 9.17E-06 | 0.00395844 | 0.56416079 | 2.54E-03 | 0.44244989 |
| **120** | Ifi211 | 2.57932934 | 8.13E-05 | 0.01541534 | 0.44786150 | 2.93E-04 | 0.17615244 |
| **121** | Zbp1 | 3.45668201 | 9.96E-06 | 0.00409316 | 0.56806247 | 3.49E-03 | 0.48624420 |
| **122** | Nlrp3 | 2.17001642 | 7.72E-03 | 0.24767084 | 0.53729578 | 2.13E-02 | 0.84390998 |
| **123** | Ccrl2 | 3.45920493 | 1.80E-03 | 0.10681809 | 0.34440480 | 6.15E-03 | 0.59560035 |
| **124** | Calb2 | 2.56808017 | 5.27E-03 | 0.19888280 | 0.53296640 | 1.79E-02 | 0.81920148 |
| **125** | H2-Q6 | 2.02003378 | 1.54E-03 | 0.09743142 | 0.65098913 | 1.39E-02 | 0.76433382 |
| **126** | Tap1 | 1.88860122 | 3.96E-02 | 0.49213069 | 0.56880354 | 3.47E-03 | 0.22267670 |
| **127** | Cxcl9 | 2.52385191 | 5.95E-03 | 0.21332158 | 0.42123095 | 8.67E-03 | 0.67005142 |
| **128** | Ubd | 2.39896303 | 1.67E-04 | 0.02562917 | 0.44043610 | 3.19E-04 | 0.17615244 |
| **129** | Il6 | 1.93183686 | 1.22E-02 | 0.31324421 | 0.54486710 | 1.98E-02 | 0.82942416 |
| **130** | Tnfsf9 | 2.37887443 | 5.22E-03 | 0.19868864 | 0.56107327 | 3.85E-02 | 0.89732537 |
| **131** | Cxcl5 | 2.54916720 | 4.76E-04 | 0.04949381 | 0.38358909 | 2.78E-04 | 0.17615244 |
| **132** | Acod1 | 2.36666049 | 1.02E-04 | 0.01799975 | 0.50172742 | 6.87E-04 | 0.25065604 |
| **133** | Slfn5 | 2.42192532 | 7.19E-05 | 0.01435027 | 0.63131721 | 2.70E-03 | 0.44792725 |
| **134** | Parp9 | 2.62734183 | 3.55E-04 | 0.04044717 | 0.48609779 | 2.57E-03 | 0.44244989 |
| **135** | Cd83 | 1.82495088 | 4.94E-02 | 0.52528080 | 0.52640987 | 3.24E-02 | 0.87587093 |
| **136** | C3 | 2.31966649 | 8.01E-05 | 0.01531154 | 0.66479115 | 2.94E-02 | 0.86522318 |
| **137** | Gm4070 | 2.80682922 | 8.76E-06 | 0.00389854 | 0.60459103 | 3.14E-04 | 0.17615244 |
| **138** | Gadd45g | 2.29540722 | 1.35E-02 | 0.32730819 | 0.40264765 | 7.32E-03 | 0.63844047 |
| **139** | Slc15a3 | 1.70807624 | 1.84E-03 | 0.10804654 | 0.56269320 | 1.09E-02 | 0.37581889 |
| **140** | Ddx58 | 2.62244571 | 9.07E-04 | 0.07135547 | 0.59389039 | 1.09E-02 | 0.72032855 |
| **141** | Rnf213 | 4.35691671 | 1.60E-05 | 0.00550810 | 0.32752648 | 5.98E-03 | 0.59472799 |
| **142** | Plek | 3.70375270 | 2.23E-03 | 0.12127001 | 0.33522574 | 6.50E-03 | 0.60965523 |
| **143** | Samd9l | 2.25739073 | 2.58E-05 | 0.00729772 | 0.54630415 | 1.94E-04 | 0.15323624 |
| **144** | H2-T23 | 2.41236379 | 3.48E-03 | 0.15820859 | 0.59984178 | 2.99E-02 | 0.86529960 |
| **145** | Pik3ap1 | 2.21030370 | 1.11E-03 | 0.08058216 | 0.55879035 | 5.67E-03 | 0.58439418 |
| **146** | Dusp2 | 2.22053594 | 3.53E-03 | 0.15968304 | 0.65732298 | 4.03E-02 | 0.63862158 |
| **147** | Icosl | 2.14510713 | 3.38E-04 | 0.01403969 | 0.64001141 | 7.84E-03 | 0.32280108 |
| **148** | Batf | 2.59124509 | 1.68E-06 | 0.00181909 | 0.52179489 | 1.73E-04 | 0.15323624 |
| **149** | Pdlim4 | 2.26412970 | 2.55E-03 | 0.13188156 | 0.61020570 | 3.56E-02 | 0.89397250 |
| **150** | Itga5 | 2.51041433 | 2.41E-02 | 0.41718183 | 0.64120071 | 1.38E-03 | 0.33943803 |
| **151** | Ptgs2 | 2.10473239 | 9.87E-03 | 0.28130101 | 0.53531845 | 1.16E-02 | 0.73162649 |
| **152** | Vasp | 2.12858336 | 1.08E-04 | 0.01873055 | 0.57996932 | 7.26E-04 | 0.25621170 |
| **153** | Ube2l6 | 2.14718554 | 9.94E-04 | 0.07503100 | 0.58164216 | 9.35E-03 | 0.67934527 |
| **154** | Upp1 | 1.69925490 | 1.58E-02 | 0.35172316 | 0.62839167 | 1.32E-02 | 0.40327595 |
| **155** | Asap3 | 2.00700975 | 2.19E-04 | 0.03017068 | 0.62928075 | 3.61E-03 | 0.49336737 |
| **156** | Parp10 | 2.44802034 | 8.35E-05 | 0.01551513 | 0.48242969 | 8.97E-03 | 0.67031965 |
| **157** | Tiparp | 1.99802126 | 2.93E-02 | 0.44228292 | 0.65643726 | 2.68E-02 | 0.85946297 |
| **158** | Pfkfb3 | 2.16801813 | 1.56E-02 | 0.34916288 | 0.49137190 | 3.01E-02 | 0.86529960 |

| | | | | | | | |
|---|---|---|---|---|---|---|---|
| **159** | Rnd1 | 2.01686683 | 6.81E-03 | 0.23023321 | 0.41604455 | 4.41E-04 | 0.20587882 |
| **160** | Mx1 | 1.97990188 | 9.21E-03 | 0.12409455 | 0.54582184 | 1.96E-02 | 0.48629091 |
| **161** | Ccr7 | 1.97052132 | 9.59E-05 | 0.01712659 | 0.52096540 | 1.36E-04 | 0.15281927 |
| **162** | Mlkl | 1.63637365 | 4.12E-02 | 0.49606170 | 0.64549448 | 1.58E-02 | 0.44718460 |
| **163** | Myc | 2.11771656 | 2.30E-02 | 0.40803683 | 0.65743868 | 1.25E-02 | 0.73258198 |
| **164** | Helz2 | 1.94085115 | 1.62E-03 | 0.10070020 | 0.57061425 | 3.21E-03 | 0.47187986 |
| **165** | Pim1 | 1.94884581 | 1.90E-03 | 0.11001012 | 0.53289015 | 7.92E-04 | 0.26712765 |
| **166** | Lcp2 | 2.05294469 | 5.99E-06 | 0.00316439 | 0.61676437 | 1.19E-05 | 0.05332822 |
| **167** | Tapbp | 1.94845435 | 7.15E-04 | 0.06156592 | 0.64899178 | 6.31E-03 | 0.60049723 |
| **168** | Relb | 1.71072725 | 4.46E-02 | 0.50730862 | 0.37779642 | 4.16E-03 | 0.51231447 |
| **169** | Tifa | 2.69756031 | 2.77E-02 | 0.43537826 | 0.49485059 | 7.54E-03 | 0.64328431 |
| **170** | Nos2 | 1.86329351 | 7.08E-05 | 0.01419552 | 0.58505342 | 2.77E-04 | 0.17615244 |
| **171** | Samsn1 | 2.70583354 | 5.90E-04 | 0.05478513 | 0.50369259 | 4.05E-03 | 0.51186592 |
| **172** | Il19 | 1.58919929 | 3.54E-02 | 0.47611167 | 0.63907034 | 3.48E-02 | 0.60266886 |
| **173** | Rtp4 | 1.84952382 | 1.16E-02 | 0.30394537 | 0.59052521 | 1.55E-02 | 0.79734984 |
| **174** | Ifih1 | 1.86774876 | 4.43E-03 | 0.07741710 | 0.57886267 | 1.76E-02 | 0.47222336 |
| **175** | Irak2 | 1.86555067 | 4.86E-03 | 0.08211376 | 0.64688485 | 5.79E-03 | 0.28598585 |
| **176** | Ccl19 | 1.66544189 | 1.36E-03 | 0.09108459 | 0.65673172 | 2.56E-02 | 0.52788157 |
| **177** | Dhx58 | 1.93044384 | 8.65E-03 | 0.26111278 | 0.63803224 | 4.18E-02 | 0.90189594 |
| **178** | Mcoln2 | 1.68976262 | 1.23E-03 | 0.08588359 | 0.64034120 | 2.88E-04 | 0.09077273 |
| **179** | Mmp13 | 1.77795373 | 2.15E-05 | 0.00666258 | 0.61078982 | 1.73E-04 | 0.15323624 |
| **180** | Rhoc | 1.73409894 | 1.30E-02 | 0.15240257 | 0.25466171 | 3.81E-02 | 0.89732537 |
| **181** | Ptafr | 1.71360615 | 3.61E-04 | 0.04080149 | 0.57218963 | 2.44E-04 | 0.16598038 |
| **182** | Tm4sf1 | 1.71116246 | 7.30E-04 | 0.02311345 | 0.62841316 | 1.45E-03 | 0.15018223 |
| **183** | Tnip1 | 3.46312363 | 5.70E-04 | 0.05371134 | 0.38419003 | 2.62E-02 | 0.85803933 |
| **184** | Fosl1 | 1.68963080 | 7.58E-04 | 0.06317860 | 0.65535762 | 4.45E-03 | 0.52607158 |
| **185** | Trib1 | 1.76398456 | 3.05E-02 | 0.44983005 | 0.59167321 | 1.94E-03 | 0.39419250 |
| **186** | Olfr56 | 1.67901283 | 4.65E-03 | 0.07990582 | 0.65962279 | 1.85E-02 | 0.47484463 |
| **187** | Csf1 | 3.84986473 | 3.87E-03 | 0.16826366 | 0.65253596 | 1.42E-03 | 0.15018223 |
| **188** | Ifi203 | 1.65665843 | 3.99E-03 | 0.07148342 | 0.48393181 | 2.93E-02 | 0.86522318 |
| **189** | Gem | 2.18708421 | 1.45E-02 | 0.33877419 | 0.50281949 | 1.23E-02 | 0.73162649 |
| **190** | Adamts4 | 1.59600272 | 7.87E-03 | 0.24944666 | 0.45218585 | 2.63E-05 | 0.08436031 |
| **191** | Sp100 | 1.79040760 | 4.37E-03 | 0.18053542 | 0.50307017 | 2.06E-03 | 0.40708002 |
| **192** | Mndal | 1.52522924 | 1.23E-03 | 0.03275990 | 0.48249225 | 1.48E-02 | 0.77969562 |
| **193** | Steap1 | 1.56662354 | 2.00E-03 | 0.11408367 | 0.61142439 | 2.33E-04 | 0.16556984 |
